# Clinical and Psychosocial Predictors of Physical Activity in Systemic Lupus Erythematosus: A Multicentre Cross-Sectional Study

**DOI:** 10.3390/healthcare13212768

**Published:** 2025-10-31

**Authors:** Alba Castañón-Fernández, Rubén Cuesta-Barriuso, José María Torres-Quiles

**Affiliations:** 1 Faculty of Medicine and Health Sciences, University of Oviedo, 33006 Oviedo, Spain; albaaacf@gmail.com; 2 Department of Surgery and Medical-Surgical Specialties, University of Oviedo, 33006 Oviedo, Spain; 3 InHeFis Research Group, Instituto Asturiano de Investigación Sanitaria (ISPA), 33011 Oviedo, Spain; 4 Department of Physiotherapy, University of Murcia, 30120 Murcia, Spain; jm.torresquiles@um.es

**Keywords:** systemic lupus erythematosus, motor activity, physical activity, fatigue, quality of life, sleep–wake disorders, depression, anxiety

## Abstract

**Highlights:**

**What are the main findings?**

- In patients with systemic lupus erythematosus, physical activity was significantly associated with sleep quality, anxiety, and perceived physical health.
- Better physical functioning was related to higher activity levels, while an unexpected association was observed between poorer sleep quality and greater physical activity.

**What are the implications of the main findings?**

- These findings highlight the need to integrate psychosocial and behavioural factors into the assessment and promotion of physical activity in lupus, beyond conventional clinical variables.
- They support the development of tailored, supervised exercise programs that address anxiety and sleep disturbances as part of multidisciplinary care in systemic lupus er-ythematosus.

**Abstract:**

**Background/Objectives**: Systemic lupus erythematosus (SLE) is a chronic autoimmune disease characterised by musculoskeletal manifestations such as myopathies, arthritis, and arthralgia. Physical activity may improve patients’ quality of life and overall wellbeing. This study aimed to evaluate physical activity levels in patients with SLE and identify how clinical, psychosocial, and sociodemographic factors influence these levels. **Methods**: A multicentre cross-sectional study was conducted including 64 patients with SLE. Clinical variables were obtained from medical records, and patient-reported outcomes were collected at the time of the survey. Physical activity was assessed using the International Physical Activity Questionnaire (IPAQ-SF). Independent variables included fatigue (FAS), quality of life (SF-36), sleep (PSQI), depression (BDI-II), anxiety (HARS), age, disease factors (activity, duration, damage), sex, smoking, and comorbidities. **Results**: Significant associations were found between physical activity levels and smoking status (χ^2^ = 11.88; *p* = 0.003), sleep quality (χ^2^ = 6.81; *p* = 0.03), and anxiety (χ^2^ = 18.39; *p* = 0.001). In multivariable analyses, poor sleep (PSQI > 5) (OR = 14.40; 95% CI: 2.50–82.99), higher anxiety (HARS; per point OR = 1.12; 95% CI: 1.05–1.20), and higher SF-36 Physical Component Summary (PCS) scores (per point OR = 1.29; 95% CI: 1.15–1.45) were associated with being in a higher physical activity category. Given the counterintuitive direction for sleep and the limited model fit, these results should be interpreted cautiously. **Conclusions**: In patients with SLE, physical activity was associated with sleep disturbances, anxiety, and perceived physical health. These findings underscore the need to integrate psychosocial and behavioural factors into multidisciplinary strategies promoting physical activity in lupus care and provide a rationale for future longitudinal and interventional studies to validate and extend these associations.

## 1. Introduction

Systemic lupus erythematosus (SLE) is a chronic autoimmune disease with multi-organ involvement, most commonly affecting the skin, kidneys, and joints [1]. The aetiology of SLE remains elusive, and its clinical heterogeneity continues to pose substantial diagnostic challenges [2]. The clinical course and prognosis vary considerably, with potentially severe outcomes, including early mortality [3]. Typical manifestations include pleuritis, malar rash, alopecia, arthritis, and lupus nephritis [1].

Antimalarials, particularly hydroxychloroquine, are widely used and have been shown to reduce flare frequency and thrombosis risk, while improving cutaneous and musculoskeletal symptoms, as well as overall survival. Glucocorticoids and biologics such as Belimumab^®^, Rituximab^®^, Anifrolumab^®^, and Voclosporin^®^ are also commonly prescribed [4].

SLE predominantly affects women of reproductive age, with a female-to-male ratio of 9:1 [5], likely due to hormonal, genetic, epigenetic, and environmental factors [6]. Patients often develop comorbidities, most notably cardiovascular disease and severe infections, which adversely affect prognosis and survival [7]. Age at diagnosis influences disease expression; juvenile-onset SLE (<18 years) tends to follow a more aggressive course, with higher disease activity, greater treatment burden, and increased morbidity and mortality [8].

Disease activity and organ damage are key clinical parameters. Disease activity refers to reversible clinical or laboratory findings reflecting current immunological or inflammatory processes [9]. Studies suggest an inverse association between disease activity and health-related quality of life (HRQoL) [10]. By contrast, organ damage is irreversible and results from cumulative inflammation, treatment side effects, or comorbidities [11]. It predicts long-term morbidity, mortality, and healthcare costs [12], with higher damage scores associated with lower socioeconomic status, African descent, older age, and longer disease duration [13,14].

Physical activity levels in patients with SLE are typically low, correlating with increased cardiovascular risk and mortality [15]. Aerobic exercise (e.g., walking, jogging) has been shown to reduce fatigue and depressive symptoms without exacerbating disease activity [16]. Sleep quality is also markedly impaired in SLE and is associated with heightened disease activity, cardiovascular complications, and depressive symptoms [16].

Fatigue is one of the most common and disabling symptoms in SLE, significantly impairing HRQoL and work capacity. Contributing factors include physical inactivity, psychological stressors, pain, and fibromyalgia [17]. Patients with SLE consistently report lower HRQoL compared with healthy controls, largely due to medication side effects and multisystem involvement [18].

Psychosocial symptoms, particularly depression and anxiety, are highly prevalent. Depression is linked to greater disability and poorer treatment adherence, underscoring the importance of early identification [19]. Anxiety likewise reduces HRQoL and interferes with daily functioning, often reinforcing a more negative illness perception [20]. Given the limited and heterogeneous evidence, the present work was conceived as an exploratory, pilot, and hypothesis-generating study to identify preliminary clinical and psychosocial correlates for future confirmatory research.

Although research on physical activity in SLE has increased in recent years, most studies have focused on clinical or physiological aspects, with limited integration of psychosocial and demographic factors [21,22,23,24]. This relative paucity of multidimensional approaches restricts understanding of how diverse determinants jointly influence patients’ engagement in regular physical activity and their overall functional outcomes. Addressing this limitation is clinically relevant, as incorporating psychosocial and behavioural variables into clinical decision-making may enhance exercise adherence, fatigue management, and quality of life.

Despite increasing evidence on the safety and physiological benefits of physical activity in systemic lupus erythematosus, previous research has largely overlooked the psychosocial and clinical determinants that influence patients’ engagement in physical activity. Most studies have focused on disease activity, fatigue, or cardiorespiratory outcomes, with limited attention to behavioural and emotional factors such as anxiety, sleep quality, or perceived health status. Understanding how these dimensions interact is crucial to designing comprehensive, patient-centred physical activity strategies. Addressing this gap, the present study explores the clinical, psychosocial, and sociodemographic correlates of physical activity in individuals with systemic lupus erythematosus.

This study aimed to evaluate physical activity levels in patients with SLE and to examine multivariable associations with clinical, psychosocial, sociodemographic, and anthropometric variables. Understanding these associations may help inform the development of tailored intervention programmes and multidisciplinary strategies to promote physical activity and improve overall health outcomes in patients with systemic lupus erythematosus.

## 2. Materials and Methods

### 2.1. Study Design

This was a multicentre cross-sectional study. Accordingly, the study was designed as an exploratory, hypothesis-generating investigation aimed at identifying preliminary associations rather than establishing causal relationships. The ambispective design comprised retrospective collection of clinical data from medical records and prospective assessment through self-administered questionnaires. The retrospective component referred exclusively to the extraction of previously documented clinical information, whereas the prospective component focused on the administration of patient-reported outcome measures at the time of data collection. For clarity, throughout this study the term “physical activity” refers to any habitual bodily movement that results in energy expenditure, as assessed by the International Physical Activity Questionnaire (IPAQ). The term “exercise” is used only when referring to structured or planned training programmes described in the previous literature. The study was designed and reported in accordance with the Strengthening the Reporting of Observational Studies in Epidemiology (STROBE) guidelines for cross-sectional studies [25].

### 2.2. Ethical Considerations

Confidentiality and anonymity of all data were guaranteed in accordance with the ethical principles of the Declaration of Helsinki of the World Medical Association. Written informed consent was obtained from all participants prior to questionnaire completion.

The study protocol was approved by the Research Ethics Committee of the Principality of Asturias (CEImPA 2025.042) and registered in the international clinical trial registry ClinicalTrials.gov (NCT06919250).

### 2.3. Participants

Inclusion criteria: (i) confirmed diagnosis of systemic lupus erythematosus (SLE); (ii) adults (≥18 years); (iii) both sexes; and (iv) provision of written informed consent.

Exclusion criteria: (i) individuals dependent on others for basic activities of daily living; (ii) those unable to ambulate independently; and (iii) those who had undergone surgical procedures within the previous 6 months.

### 2.4. Study Variables

The primary variable was the level of physical activity in patients with SLE. Secondary and modifying variables included fatigue, health-related quality of life, sleep disturbances, depressive and anxiety symptoms, age, and disease activity, duration, and damage. Potential confounders were sex, smoking status, comorbidities, and treatment exposure. The main assessment tools were:

- Physical activity: assessed using the International Physical Activity Questionnaire (IPAQ) [26], a 7-item tool designed to evaluate activity levels in adults aged 18–65. It measures frequency, duration, and intensity of physical activity over the preceding week, including time spent walking and sitting. Results are categorised into low, moderate, or high activity levels, reflecting compliance with WHO recommendations [27]. Outcomes are expressed in metabolic equivalent units (METs), with higher MET values indicating greater physical activity.
- Health-related quality of life: measured using the Spanish version of the Short Form-36 Health Survey (SF-36) [28]. This 36-item questionnaire covers eight domains: physical functioning, role physical, bodily pain, general health, vitality, social functioning, role emotional, and mental health, including a transition item. Scores range from 0 to 100, with higher scores reflecting better perceived quality of life.
- Fatigue: assessed using the Fatigue Assessment Scale (FAS), a 10-item instrument measuring physical and mental fatigue [29]. Scores range from 10 to 50, with higher scores indicating more severe fatigue.
- Sleep quality: evaluated with the Pittsburgh Sleep Quality Index (PSQI), a 19-item questionnaire comprising seven components: subjective sleep quality, latency, duration, efficiency, disturbances, use of sleep medication, and daytime dysfunction [30]. Scores range from 0 to 21, with values > 5 indicating poor sleep quality.
- Depressive symptoms: assessed with the Beck Depression Inventory-II (BDI-II), a 21-item instrument covering emotional, cognitive, motivational, and physiological domains [31]. Total scores range from 0 to 63, with scores ≥ 15 suggesting probable clinical depression.
- Anxiety symptoms: measured using the Hamilton Anxiety Rating Scale (HARS), a 14-item instrument assessing both somatic and psychological anxiety symptoms [32]. Scores range from 0 to 56 and are categorised as follows: no/mild anxiety (<17), mild to moderate (18–24), and moderate to severe (>25).

Sociodemographic variables collected included age, sex, and smoking status. Clinical variables included disease activity, duration, and damage, as well as current pharmacological treatment. Disease activity and organ damage were self-reported by patients on the basis of prior medical information, without direct clinical verification. This approach may lead to limited reliability and comparability versus validated clinical indices (e.g., SLEDAI, SLICC/ACR-DI), and therefore represents a methodological limitation that should be considered when interpreting the findings. These variables were not assessed using standardised indices such as the Systemic Lupus Erythematosus Disease Activity Index (SLEDAI) or the Systemic Lupus International Collaborating Clinics/American College of Rheumatology Damage Index (SLICC/ACR DI), which restricts comparability with studies employing validated clinical measures. This choice reflected the remote, patient-centred design prioritising patient-reported outcomes (PROs) and precluded contemporaneous clinical verification at the time of survey completion. The selection of these assessment tools was based on their established psychometric reliability, availability of validated Spanish versions, and extensive use in clinical and epidemiological research involving patients with systemic lupus erythematosus and other rheumatic diseases. Their widespread application supports the methodological validity and comparability of the present findings with the prior literature.

### 2.5. Procedure

Data were collected between April and May 2025 using a structured, closed-ended questionnaire. No identifying information (e.g., name, ID) was obtained. Participants were recruited through two patient associations (the Lupus Association of Asturias and the Autoimmune and Lupus Association of Almería). While this facilitated access to a well-characterised population, it may have introduced a selection bias, as individuals affiliated with patient organisations are often more proactive in managing their health and may report higher engagement in physical activity than the broader SLE population. To mitigate this potential bias, participation was voluntary and anonymous and was open across several Spanish regions, which may have improved representativeness within the available sample. This potential selection effect should be considered when interpreting the findings.

Researchers contacted lupus patient associations across several Spanish regions. After providing study details, a summary presentation was disseminated via the associations’ internal channels and social media platforms for patient outreach. Survey data were analysed under the supervision of the academic advisor, in compliance with data protection regulations, ensuring full anonymity of clinical information and patient identity. Because several clinical variables (e.g., disease activity and organ damage) were self-reported by participants, there is a potential risk of recall or interpretation bias. To minimise this, the survey was designed using clear, structured questions with non-technical terminology derived from prior clinical documentation, and participants were informed that their responses would remain fully anonymous. These procedures aimed to enhance comprehension and reduce social desirability or reporting bias, thereby improving the overall reliability of the self-reported data.

### 2.6. Sample Size

A recent systematic review estimated that 11–29.8% of SLE patients meet WHO physical activity recommendations [23]. Assuming an expected prevalence of 20.4% (*p* = 0.20, SD = 0.40), a precision of ±10%, and a 95% confidence level, the required sample size was calculated as 63 patients. The calculated sample size (n = 63) was fully achieved, which reinforces the internal validity of the analyses despite the limited sample size.

### 2.7. Statistical Analysis

All analyses were conducted using IBM SPSS Statistics v26. Descriptive statistics for quantitative variables included means and standard deviations. Normality was assessed with the Shapiro–Wilk test. Several variables deviated from normality, justifying the use of non-parametric tests. To address potential issues of sparse cells in categorical analyses, sensitivity analyses were performed by collapsing categories with low frequencies (e.g., smoking status, anxiety levels) and by applying exact tests (Fisher’s exact test) when appropriate. These procedures were used to verify the robustness of the chi-square results. Categorical variables were summarised as absolute and relative frequencies (percentages).

Physical activity and psychosocial variables were coded according to standard scoring manuals. Associations between physical activity levels (low, moderate, high) and clinical, sociodemographic, and psychosocial variables were examined using chi-square tests for categorical variables and Kruskal–Wallis tests for non-normally distributed quantitative variables.

Spearman’s rank correlation was applied to assess associations involving continuous variables. An ordinal logistic regression model was then developed to identify associations between physical activity levels and clinical, psychosocial, and quality of life variables showing statistical significance in preliminary analyses. Given the suboptimal model fit and the low expected counts in several cells, further validation in larger cohorts or the application of alternative modelling strategies (e.g., binary logistic regression or multilevel models) is warranted.

Potential confounding variables such as age, sex, comorbidities, and pharmacological treatment were assessed for collinearity and included in the regression model if deemed clinically or statistically relevant. We coded physical activity as an ordinal outcome (Low/Moderate/High). The proportional odds assumption was assessed (e.g., Brant test); results are reported in the Appendix A tables. There were no missing data for the primary and secondary variables. A *p*-value of α < 0.05 was considered statistically significant in all analyses.

## 3. Results

A total of 64 patients with systemic lupus erythematosus (SLE) completed the questionnaires. The mean age was 50.99 years (SD = 12.82), and the average disease duration since diagnosis was 16.76 years (SD = 11.52). Most participants were female (84.4%), and 57.8% lived with a partner. Only 53.1% reported being aware of their disease activity and organ damage. Comorbidities were present in 60.9%, while 12.5% were current smokers. Descriptive statistics are shown in Table 1.

A statistically significant association was observed between physical activity level and smoking status (χ^2^ = 11.88; *p* = 0.003), sleep disturbances (χ^2^ = 6.81; *p* = 0.03), and anxiety category (χ^2^ = 18.39; *p* = 0.001). For smoking status and anxiety, several expected counts were <5; therefore, these results should be interpreted with caution (Table 2). Detailed observed and expected cell frequencies are provided in the Appendix A (Table A1).

Sensitivity analyses, using collapsed categories for sparse variables and Fisher’s exact test, yielded results consistent with the primary chi-square findings, confirming the direction and statistical significance of the associations (see Table A2). 

Kruskal–Wallis tests showed that physical activity level (IPAQ categories derived from MET-min/week) was significantly associated with several SF-36 domains. Participants with higher physical activity reported better scores in Physical Function (H = 8.61, *p* = 0.013, ε^2^ = 0.11), Vitality (H = 8.13, *p* = 0.02, ε^2^ = 0.10), and Mental Health (H = 8.29, *p* = 0.02, ε^2^ = 0.10). Significant associations were also observed for the Physical Component Summary (PCS; H = 9.70, *p* = 0.01, ε^2^ = 0.13) and the Mental Component Summary (MCS; H = 7.76, *p* = 0.02, ε^2^ = 0.09). Effect sizes were small-to-moderate (Table 3). Although most of the effect sizes estimated using epsilon-squared (ε^2^) were in the small-to-moderate range, these results should not be disregarded. In systemic lupus erythematosus, even modest differences in physical or mental health indicators can have meaningful clinical implications, as slight impairments in fatigue, vitality, or perceived physical functioning may significantly affect daily activities and quality of life. Therefore, the observed statistical effects, while limited in magnitude, highlight potentially relevant clinical variations that warrant attention in multidisciplinary care and future longitudinal research.

Spearman’s correlation analysis showed that higher weekly MET-min were positively associated with depressive symptoms (ρ = 0.35; *p* = 0.01), anxiety (ρ = 0.40; *p* < 0.001), and physical functioning (ρ = 0.26; *p* = 0.04). Conversely, MET-min were inversely associated with the SF-36 Mental Health domain (ρ = −0.29; *p* = 0.02) and the Mental Component Summary (ρ = −0.25; *p* = 0.04). No other significant correlations were identified (*p* > 0.05; Table 4). These correlations indicate that higher levels of physical activity were modestly associated with better perceived physical functioning and with greater self-reported anxiety and depressive symptoms. This pattern may reflect that more active patients have greater awareness of their physical and emotional status, or that physical activity could function as a behavioural coping strategy in those experiencing psychological distress. However, given the cross-sectional design and the small-to-moderate strength of the correlations, these associations should be interpreted cautiously and cannot imply causality. 

These correlations in SLE may reflect bidirectional mechanisms. Greater physical activity could help reduce anxiety via physiological and psychological pathways, whereas individuals with higher anxiety may engage in physical activity as a coping strategy. This complexity reinforces the need for longitudinal studies to clarify causal direction. Clinically, changes in total MET-min/week may represent meaningful improvements in daily functioning and health behaviour. Higher activity levels can indicate greater autonomy, improved activity tolerance, and more effective coping with fatigue, anxiety, or stress. Even modest increases in physical activity may yield tangible benefits for both physical and psychosocial wellbeing in SLE.

These divergent patterns indicate that the relation between PA and mental health may be non-linear, highlighting the importance of modelling PA both categorically and continuously in future studies.

In cross-tabulations, poor sleep was more frequent among participants in the high PA category than in the low PA category (see Appendix A Table A1), consistent with the positive adjusted association of PSQI > 5 with higher PA in the ordinal model. Although statistically significant, this counterintuitive pattern should be interpreted in the context of sparse contingency cells and the temporal mismatch between IPAQ (7-day recall) and PSQI (1-month recall).

The ordinal logistic regression model (higher values indicating higher physical activity) identified significant associations with poor sleep (OR = 14.40; 95% CI: 2.50–82.99; *p* = 0.003), anxiety (HARS, per-point OR = 1.12; 95% CI: 1.05–1.20; *p* < 0.001), and the SF-36 Physical Component Summary (per-point OR = 1.29; 95% CI: 1.15–1.45; *p* < 0.001). Model fit was limited (sample size relative to predictors and sparse contingency), and findings should be considered exploratory. Variance inflation factors were below conventional thresholds for problematic multicollinearity (all VIF < 5). Full regression results are presented in Table 5.

In the ordinal regression model, poorer sleep quality (higher PSQI scores), higher anxiety levels, and better physical functioning (SF-36 PCS) were significantly associated with higher categories of physical activity. These results suggest that patients reporting more anxiety or sleep disturbances, as well as those with better physical health perception, tended to engage in more physical activity. Although these associations were statistically significant, they should be interpreted cautiously due to the small sample size (n = 64) and the moderate model fit (Nagelkerke R^2^ = 0.29). The limited number of predictors relative to the sample size may have increased the risk of overfitting, and therefore the observed relationships require confirmation in larger, prospectively designed studies.

## 4. Discussion

This study aimed to evaluate physical activity levels in adults with systemic lupus erythematosus (SLE) and to examine their associations with clinical, psychosocial, and sociodemographic variables, as well as to identify the strongest multivariate associations of physical activity in this population. Our findings reveal a heterogeneous distribution of physical activity levels among patients with SLE, with statistically significant associations with poor sleep quality, counterintuitive associations with anxiety, and higher physical health-related quality of life.

Contrary to expectations, poor sleep quality was positively associated with higher physical activity levels. This counterintuitive finding may reflect self-report or selection biases, reverse causality (e.g., some patients engaging in physical activity as a coping strategy for sleep problems), or mismatched recall periods between the IPAQ (7-day) and PSQI (1-month) time frames. These differences could have contributed to paradoxical associations, as variations in daily activity may not align with longer-term sleep patterns. Although the model fit was limited, these results should be interpreted cautiously and considered exploratory rather than causal.

Nonetheless, prior evidence supports a bidirectional relationship between physical activity and sleep. Lin et al. [33] reported that a four-week aerobic programme improved sleep quality in SLE, while a recent meta-analysis confirmed that non-pharmacological interventions, including structured physical activity, improve fatigue, depression, and quality of life in this population [34]. Furthermore, the 2024 EULAR recommendations emphasise physical activity as a core element of non-pharmacological SLE management, supporting its role in maintaining function and psychosocial wellbeing [21].

These observations, together with the cross-sectional nature of our data, suggest that tailored and supervised physical activity programmes may improve sleep quality and help alleviate anxiety in SLE. Future research should include objective assessment methods (e.g., actigraphy, wearable monitoring) to reduce recall bias. From a clinical perspective, these findings also support the hypothesis that physical activity could act as a behavioural coping mechanism for anxiety or sleep disturbances, transforming counterintuitive results into potentially meaningful insights. Similar behavioural mechanisms have been described in chronic disease populations, where physical activity and sleep interact bidirectionally [35].

Unexpectedly, higher anxiety scores were positively correlated with greater physical activity in our sample. This finding may reflect self-report patterns or sample-specific effects, although previous evidence supports the beneficial role of physical activity in reducing anxiety, depression, and fatigue in SLE [36]. These associations should therefore be interpreted as exploratory within the methodological limitations of the study.

A positive perception of physical functioning appears to facilitate greater engagement in an active lifestyle among individuals with chronic illness. In our study, higher scores on the SF-36 physical component summary were significantly associated with greater physical activity levels. These findings are consistent with prior evidence from tele-exercise interventions, in which participants with SLE demonstrated clinically meaningful improvements in both physical and emotional role functioning [37]. Although several SF-36 domains, including vitality and mental health, showed significant bivariate associations with physical activity levels, these variables did not retain statistical significance in the multivariate model. This suggests that their influence may be mediated by other factors, such as perceived physical functioning or psychological symptoms.

Notably, associations differed when physical activity was analysed as categorical IPAQ levels versus continuous MET-min/week. While higher IPAQ categories related to better SF-36 Mental Health in Kruskal–Wallis tests, MET-min/week showed small inverse correlations with Mental Health and MCS. This divergence suggests non-linear or threshold effects, and/or distributional influences (e.g., influential high-MET observations). Future work should model non-linearity (e.g., restricted cubic splines) and predefine physical activity contrasts to clarify dose–response patterns.

Notably, associations differed when physical activity was analysed as categorical versus continuous variables. No associations were found between physical activity and either age or disease duration, consistent with previous research reporting heterogeneous results among SLE populations [38,39]. Evidence regarding the influence of physical activity on SLE disease activity remains inconsistent, with studies reporting both improvements and null effects across different exercise modalities [23,40,41].

Fatigue and depressive symptoms are well documented in SLE and represent major barriers to sustaining physical activity. In this study, depressive symptoms showed an unexpected positive correlation with activity levels, which may reflect self-reporting patterns or greater disease awareness among more active patients. Fatigue scores were not significantly correlated with physical activity, possibly due to the limited sample size or psychosocial factors influencing motivation and perceived capacity. Nevertheless, fatigue is consistently cited as one of the main barriers to physical activity in this population [17]. Evidence from recent reviews confirms that structured aerobic and resistance training programmes can alleviate fatigue, reduce depressive symptoms, and improve overall quality of life in individuals with inflammatory rheumatic and musculoskeletal diseases, including SLE [23,34,42].

Integrating supervised physical activity counselling with sleep and anxiety management may be a feasible component of multidisciplinary SLE care. Adequately powered longitudinal studies are needed to confirm its long-term effects on physical and psychosocial outcomes. These findings have relevant clinical implications. Understanding how physical activity interacts with psychosocial factors such as anxiety, sleep quality, and coping behaviour may support the design of programmes tailored to patients’ psychological needs and daily limitations. Routine assessment of these variables could guide adjustments in exercise intensity, timing, and support, enhancing adherence, perceived benefits, and quality of life in SLE.

Nevertheless, external validity is limited by the cross-sectional design and by recruitment through patient associations, which may not represent the full diversity of the SLE population. Prospective longitudinal and interventional studies are needed to confirm these associations, establish causality, and assess long-term clinical implications. Multicentre and cross-cultural collaborations will help validate and extend these findings.

### Study Limitations

Despite the study’s methodological strengths, several limitations must be acknowledged. Although the sample size met the a priori requirements, it remains relatively small for robust external validity and may have reduced statistical precision, limiting the detection of subtle associations. Given the number of independent variables in the regression, the sample may also have been insufficient to fully avoid overfitting, potentially reducing the stability of estimates. Nonetheless, the sample size was determined a priori through statistical estimation and was achieved in full, supporting the internal validity of the observed associations despite the limited number of participants. These regression findings should be considered exploratory, requiring confirmation in larger cohorts to ensure robustness. Limited model fit suggests potential unmeasured influences on physical activity in SLE, and the high odds ratio for poor sleep quality likely reflects sparse contingency patterns, warranting cautious interpretation until validated independently.

The predominance of women in the sample may have introduced sex-related bias and limited the ability to assess sex-based differences in physical activity. Additionally, the cross-sectional design precludes causal inference between the analysed predictors and physical activity outcomes.

Although validated instruments were employed, reliance on self-reported data —particularly regarding disease activity and organ damage— may have introduced reporting bias and reduced the reliability of these clinical variables. Furthermore, the recruitment of participants through patient associations may have introduced a selection bias, as these individuals are typically more health-conscious, proactive, or engaged in disease management, which may limit the generalisability of the findings to the broader SLE population. The absence of direct clinical verification at the time of data collection limits the robustness of the findings related to disease status. The lack of standardised clinical indices (e.g., SLEDAI, SLICC/ACR DI) limits comparability with studies using clinician-based assessments. Prioritising patient-reported outcomes (PROs) allowed us to capture functional, emotional, and behavioural perceptions beyond clinical markers, providing valuable complementary insight. However, differing recall periods between the instruments (IPAQ: 7 days; PSQI: 1 month) may have introduced confounding and contributed to inconsistencies in the physical activity–sleep association. Future research should integrate objective clinical indices with validated PROs to improve robustness and generalisability. 

Although the ordinal logistic regression model showed reasonable explanatory power, its suboptimal fit due to sparse data and empty cells limits generalisability and underscores the need for validation in larger, more diverse SLE cohorts. Recruitment through patient associations may have led to self-selection bias, underrepresenting individuals with greater disability or reduced access to care. The cross-sectional design further precludes causal interpretation of associations between anxiety, sleep disturbances, and physical activity. In addition, the lack of validated disease activity and damage indices (e.g., SLEDAI, SLICC/ACR DI) restricts comparability with clinician-based studies. Sparse contingency patterns in some χ^2^ tests also suggest that exact methods or category collapsing should be considered in future research to improve robustness. In addition, we conducted supplementary sensitivity analyses (Table A2) in which categorical variables with sparse distributions were reclassified into broader categories and re-evaluated using Fisher’s exact test. The results remained consistent, supporting the stability of the main associations. Moreover, the temporal mismatch between IPAQ (7 days) and PSQI (1 month) may partly contribute to the counterintuitive PA–sleep association. Moreover, the Spanish and association-based recruitment limits the generalisability of these findings to SLE populations from other healthcare systems and cultural contexts

## 5. Conclusions

This study highlights the psychosocial determinants of physical activity in systemic lupus erythematosus, supporting the integration of behavioural and emotional factors into multidisciplinary care. Physical activity was independently associated with sleep disturbances, perceived physical health, and higher anxiety. These findings should be interpreted cautiously due to the small sample size and cross-sectional design, which limit causal inference and generalisability. Larger multicentre longitudinal studies are required to validate these associations and evaluate the effects of structured physical activity interventions on psychosocial and clinical outcomes using both objective and patient-reported measures.

## Figures and Tables

**Table 1:** Descriptive statistics and distribution of sociodemographic, clinical, and psychosocial variables in patients with systemic lupus erythematosus.

Variables	Value
Age, years	50.99 ± 12.82
Disease duration, years	16.76 ± 11.52
Female sex	54 (84.4%)
Living with a partner	37 (57.8%)
Current smoker	8 (12.5%)
Comorbidities (any)	39 (60.9%)
Physical activity level (IPAQ): Low	15 (23.4%)
Physical activity level (IPAQ): Moderate	23 (35.9%)
Physical activity level (IPAQ): High	26 (40.6%)
Poor sleep quality (PSQI > 5)	24 (37.5%)
Depression category (BDI-II): Minimal	38 (59.4%)
Depression category (BDI-II): Mild	8 (12.5%)
Depression category (BDI-II): Moderate	10 (15.6%)
Depression category (BDI-II): Severe	8 (12.5%)
Anxiety category (HARS): None/Mild	26 (40.6%)
Anxiety category (HARS): Mild–Moderate	15 (23.4%)
Anxiety category (HARS): Moderate–Severe	23 (35.9%)

Values are mean ± SD for continuous variables and n (%) for categorical variables. IPAQ: International Physical Activity Questionnaire; PSQI: Pittsburgh Sleep Quality Index; BDI-II: Beck Depression Inventory-II; HARS: Hamilton Anxiety Rating Scale; MET: metabolic equivalent of task (reported as MET-min/week).

**Table 2:** Associations between physical activity levels (IPAQ categories) and categorical variables.

Variable	χ^2^	df	*p*-Value	Cramer’s V	Sparsity
Smoking status	11.88	2	0.00	0.43	50.0% cells < 5
Poor sleep (PSQI > 5)	6.81	2	0.03	0.33	0.0% cells < 5
Anxiety category (HARS)	18.39	4	0.00	0.38	11.1% cells < 5
Depression category (BDI-II)	9.29	6	0.16	0.27	75.0% cells < 5

χ^2^ = Pearson’s chi-square; df = degrees of freedom; Cramer’s V = effect size; “Sparsity” = % of cells with expected frequency <5. PSQI: Pittsburgh Sleep Quality Index; BDI-II: Beck Depression Inventory-II; HARS: Hamilton Anxiety Rating Scale.

**Table 3:** Differences in SF-36 domains and component summaries according to physical activity levels (IPAQ categories derived from MET-min/week).

Variable	H Statistic	df	*p*-Value	ε^2^
Physical Function	8.61	2	0.01	0.11
Vitality	8.13	2	0.02	0.10
Mental Health	8.29	2	0.02	0.10
Physical Component Summary (PCS)	9.70	2	0.01	0.12
Mental Component Summary (MCS)	7.76	2	0.02	0.09

H = Kruskal–Wallis statistic; df = degrees of freedom; ε^2^ = epsilon-squared effect size; PCS: Physical Component Summary; MCS: Mental Component Summary. Higher scores indicate better health status. Statistical significance set at *p* < 0.05 (two-sided).

**Table 4:** Spearman’s rank correlations between weekly MET-min and psychosocial/quality-of-life variables.

Variable	ρ (Spearman)	*p*-Value
Fatigue (FAS total)	0.12	0.33
Depression (BDI total)	0.35	0.01
Anxiety (HARS total)	0.40	0.00
Physical Function (SF-36)	0.26	0.04
Vitality (SF-36)	−0.03	0.81
Mental Health (SF-36)	−0.29	0.02
Physical Component Summary (PCS)	0.19	0.14
Mental Component Summary (MCS)	−0.25	0.04

ρ = Spearman’s rank correlation coefficient; FAS: Fatigue Assessment Scale; BDI: Beck Depression Inventory; HARS: Hamilton Anxiety Rating Scale; PCS: SF-36 Physical Component Summary; MCS: SF-36 Mental Component Summary. Negative coefficients indicate inverse associations.

**Table 5:** Ordinal logistic regression for physical activity levels (IPAQ categories derived from MET-min/week).

Predictor	OR	95% CI (Lower–Upper)	*p*-Value
Poor sleep (PSQI)	14.40	2.50–82.99	0.00
Anxiety (HARS, per point)	1.12	1.05–1.20	0.00
Physical Component Summary (PCS, per point)	1.29	1.15–1.45	0.00

OR: odds ratio; CI: confidence interval; PSQI: Pittsburgh Sleep Quality Index (poor sleep defined as PSQI > 5); HARS: Hamilton Anxiety Rating Scale (per-point continuous); PCS: SF-36 Physical Component Summary. Outcome ordered as Low < Moderate < High physical activity.

## Data Availability

The dataset generated and analysed during the current study is available in the RUO repository, University of Oviedo (doi: 10.17811/ruo_datasets.80593).

## Abbreviations

The following abbreviations are used in this manuscript:

SLE	Systemic lupus erythematosus
HRQoL	health-related quality of life
IPAQ	International Physical Activity Questionnaire
SF-36	Short Form-36 Health Survey
FAS	Fatigue Assessment Scale
PSQI	Pittsburgh Sleep Quality Index
BDI-II	Beck Depression Inventory-II
HARS	Hamilton Anxiety Rating Scale
SLEDAI	Systemic Lupus Erythematosus Disease Activity Index
SLICC/ACR DI	Systemic Lupus International Collaborating Clinics/American College of Rheumatology Damage Index
PCS	Physical Component Summary
MCS	Mental Component Summary
OR	Odds ratio
CI	Confidence interval
SD	Standard deviation

## Appendix A

**Table A1:** Observed and expected contingency tables between physical activity levels (IPAQ categories) and categorical variables in patients with systemic lupus erythematosus (n = 64).

Variable	Low PA (Obs/Exp)	Moderate PA (Obs/Exp)	High PA (Obs/Exp)
Smoking status			
Non-smoker	11/14.88	20/19.25	25/21.88
Current smoker	6/2.12	2/2.75	0/3.12
Sleep quality (PSQI)			
Good sleep	14/10.62	15/13.75	11/15.62
Poor sleep	3/6.38	7/8.25	14/9.38
Anxiety (HARS categories)			
No/mild anxiety	9/6.91	12/8.94	5/10.16
Mild–moderate anxiety	5/3.98	7/5.16	3/5.86
Moderate–severe anxiety	3/6.11	3/7.91	17/8.98
Depression (BDI categories)			
Minimal depression	12/10.09	15/13.06	11/14.84
Mild depression	3/2.12	3/2.75	2/3.12
Moderate depression	2/2.66	2/3.44	6/3.91
Severe depression	0/2.12	2/2.75	6/3.12

Obs: observed frequency; Exp: expected frequency according to Pearson’s chi-square test; PA: physical activity; IPAQ: International Physical Activity Questionnaire; PSQI: Pittsburgh Sleep Quality Index; HARS: Hamilton Anxiety Rating Scale; BDI-II: Beck Depression Inventory-II. Several cross-tabulations yielded expected cell counts <5, highlighting the need for exact tests or category collapsing in confirmatory analyses.

**Table A2:** Sensitivity Analyses for Sparse Categorical Data.

Variable (Collapsed)	Contingency Structure	*p*-Value (Two-Tailed)	ES
Smoking (Current smoker vs. Non-smoker)	High PA vs. Not-High PA × Smoking (2 × 2)	0.02	0.43
Anxiety (HARS: None–Mild–Moderate vs. Moderate–Severe)	High PA vs. Not-High PA × Anxiety (2 × 2)	0.00	0.54
Sleep (PSQI: Good vs. Poor sleep)	High PA vs. Not-High PA × Sleep (2 × 2)	0.02	0.32

Fisher’s exact test used to avoid reliance on asymptotic assumptions when expected frequencies <5; ES: Effect size (estimated using Cramer’s V).

To examine the influence of sparse cells on the robustness of chi-square results, we conducted supplementary analyses collapsing categories with low expected frequencies. Specifically, smoking status was reclassified into two groups (non-smoker vs. current smoker), and anxiety categories were collapsed into two levels (none–mild–moderate vs. moderate–severe). These collapsed variables were reanalysed using Fisher’s exact test. The direction and statistical significance of the associations with physical activity levels remained unchanged, indicating robustness of the original findings.
